# Scientometric Analysis of Public Health Emergencies: 1994–2020

**DOI:** 10.3390/ijerph19020640

**Published:** 2022-01-06

**Authors:** Jing Liu, Yujie Wang, Qian Zhang, Jianxiang Wei, Haihua Zhou

**Affiliations:** 1 School of Management, Nanjing University of Posts and Telecommunications, Nanjing 210003, China; b19111515@njupt.edu.cn (Y.W.); zhg_Qian@163.com (Q.Z.); jxwei@njupt.edu.cn (J.W.); 2 Key Research Base of Philosophy and Social Sciences in Jiangsu-Information Industry Integration Innovation and Emergency Management Research Center, Nanjing 210003, China; 3 Business School, Nanjing Xiaozhuang University, Nanjing 211171, China; zhouhaihua@njxzc.edu.cn

**Keywords:** public health emergency, CiteSpace, visual analysis, research hotspot, scientometric

## Abstract

The purpose of this paper is to summarize the research hotspots and frontiers in the field of public health emergencies (PHE) between 1994–2020 through the scientometric analysis method. In total, 2247 literature works retrieved from the Web of Science core database were analyzed by CiteSpace software, and the results were displayed in knowledge mapping. The overall characteristics analysis showed that the number of publications and authors in the field of PHE kept an upward trend during the past decades, and the United States was in the leading position, followed by China and England. Switzerland has the highest central value and plays an important intermediary role in promoting the integration and exchange of international PHE research achievements. The keyword co-occurrence analysis indicated that COVID-19 was the most high-frequency keyword in this field, and there had been no new keywords for a long time until the outbreak of COVID-19 in 2019. The burst detection analysis showed that the top five burst keywords in terms of burst intensity were zika virus, Ebola, United States, emergency preparedness and microcephaly. The results indicated that the research theme of PHE is closely related to the major infectious diseases in a specific period. It will continue to develop with more attention paid to public health. The conclusions can provide help and reference for the PHE potential researchers.

## 1. Introduction

According to Regulations on Preparedness for and Response to Emergent Public Health Hazards, Public health emergencies (PHE) refer to events that occur suddenly and cause or may cause serious damage to social and public health, such as major infectious diseases, mass unexplained diseases, major food and occupational poisoning and so on. In the era of globalization, diseases of the PHE field show the characteristics of diverse causes, more complex hazards and wider dissemination. Major public health events have a great impact on the environment, economy and even politics [1]. In 2019, the outbreak of the COVID-19 epidemic posed a great threat to people’s lives, economic development and national security, and PHE became the focus of attention of all countries. China is to improve its capacity and level of response to major public health emergencies, unswervingly implement the principle of prevention first, establish a stable mechanism for public health investment, improve the mechanism for participating in the response to international public health emergencies and promote the building of a community of human health.

Meanwhile, with the continuous development of science and technology, knowledge mapping, as a new method and new field, has achieved leapfrog development, and various excellent achievements have emerged [2]. Some scholars began to use CiteSpace as a popular knowledge mapping tool in the PHE research field. OOmwoyo pioneered the use of CiteSpace to measure the publication and dissemination of research on HIV/AIDS in Eastern or Southern Africa, analyzing the growth, productivity and scientific impact of research sources between 1980 and 2005 [3]. Tang used CiteSpace to draw the knowledge mapping of public security research, grasped the basic status and hot spots of public security research, and predicted the future development trend [4]. In view of the continuous improvement of the domestic emergency management system and the gradual realization of scientific prediction and precise prevention and control, Hu and Sun used CiteSpace software to conduct a visual analysis of PHE literature and believed that future research in the PHE field should carry out in-depth research from the aspects of theoretical development and cooperation planning [5]. Liu used CiteSpace software to process the literature data of PHE published from 2003 to 2019 and analyzed the variation trend of the number of publications, research institutions, authors and keywords, systematically understanding the global characteristics of PHE research [6].

Since the outbreak of SARS in 2003, studies and discussions on the PHE field have been increasing [7]. However, there is relatively little research on the context and visual analysis of the research hotspots and frontiers based on the scientometric method in this field. Therefore, this paper uses the CiteSpace tool to analyze the literature from 1994 to 2020 in the Web of Science Core Collection. This paper combines social network analysis, co-citation analysis and scientometrics to show research hotspots and development direction of PHE, and it also provides some constructive suggestions for the deepening development of related research on PHE. This study aims at three aspects: (1) analyzing the research status of PHE; (2) analyzing the distribution and cooperation of PHE researchers and institutions; (3) exploring the research hotspots and evolutionary context of PHE.

## 2. Materials and Methods

### 2.1. Research Tool and Method

CiteSpace is an information visualization software for analyzing co-citation networks developed by Dr. Chen Chaomei, a scholar from the school of Computer and Information Science at Drexel University. Based on co-citation analysis theory and path-finding network algorithm, the software analyzes literature citations in specific fields, draws the development trend and trend of the whole knowledge domain in a certain period, and reveals the law of scientific dynamic development. The CiteSpace tool is widely used in the research of various disciplines with powerful functions. It can provide eleven kinds of function choices to generate the maps of a co-operation network and co-occurrence. It can be used to draw the knowledge mapping of the development status and scientific structure, intuitively show the information panorama of scientific knowledge and identify core literature, hot research topics and frontier directions in a certain scientific field [8]. Through the interpretation of the maps, the interpretation function of the current situation of the field and the prediction function of the future prospect of the field are realized [9]. The software version is CiteSpace 5.7.R5 (64-bit), which is applied in this paper.

### 2.2. Data Source

CiteSpace software can analyze the literature collected by Web of Science, Scopus, PubMed and Google Scholar. Web of Science has more advantages than other databases in terms of accuracy of the journal classification system, standardization and consistency of literature records, and better visualization effect [10,11,12]. Summarizing the previous scientific measurement articles, most scholars prefer to use the Web of Science database in the bibliometric and visual analyses [13,14,15,16,17,18]. From the perspective of the analysis function, when CiteSpace conducts visual analysis, the literature data obtained from Web of Science can use all the analysis functions, while the data obtained from other databases can only use some of the analysis functions, such as domain co-occurrence analysis [19]. Therefore, this paper prefers to choose the Web of Science Core Collection (including SCI-Expanded, SSCI, CCR-Expanded and IC) as the data source database.

Considering that when investigating a rapidly growing field, the results of the topic search can obtain more reliable data sets than keyword search results, this paper used topic search [20]. The search strategy was as follows: topic search term was “public health emergenc*” (in order to ensure the comprehensiveness of data collection; word truncation algorithm “*” was used in the topic search term); the time span was from 1994 to 2020; document types were set as “Article” and “Review”. A total of 2885 initial pieces of literature were retrieved on 11 January 2021. The full information of the literature (including title, abstracts, authors, keywords, document types, journals, year of publications and the cited references) was downloaded and exported to RefWorks citation format. However, some pieces of literature lacked information, such as authors and references, so these records were excluded. Then, the exported literature information was imported into CiteSpace software for data preprocessing, such as filtering and duplication. Finally, a total of 2247 effective pieces of literature are obtained.

## 3. Results and Discussion

### 3.1. General Feature of Published Research

#### 3.1.1. Publication Years Analysis

**Figure 1:**
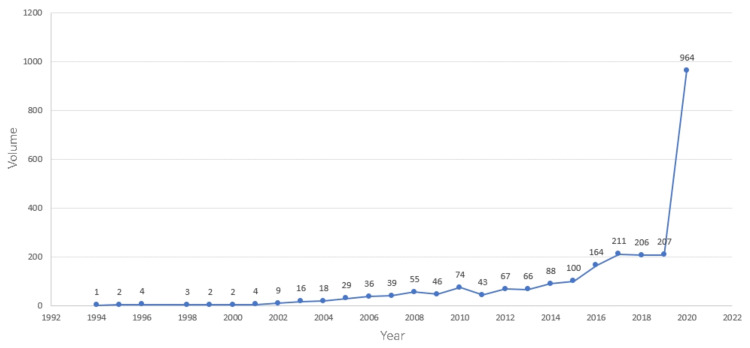
Literature volume of PHE from 1994 to 2020.

The publication of academic papers is usually regarded as an index to measure the level of the discipline’s development. The change in the number of publications can directly reflect the change in the amount of scientific knowledge [21]. Analyzing the literature published in a certain field from the perspective of time can reflect the overall trend of its research in this period to a certain extent. In the past two decades, the number of publications has increased steadily from 2003 to the peak of 964 in 2020 (see Figure 1). The research on PHE has attracted more and more attention from society and scholars. In addition, according to the growth rate of the quantity of literature, we can divide the literature quantity level into four stages. The first stage is from 1994 to 2003, the number of publications was the least. Scholars gradually began to pay attention to the field of PHE, but there were few studies and little attention. The second stage is from 2004 to 2014 when the publications were between 10 and 100, which is relatively average. It shows that the attention in this field has been appropriately improved. This is because the outbreak of SARS in 2003 raised scholars’ attention to public health events. The third stage is from 2015 to 2019 when the number of publications in each year showed an increasing trend, increasing to between 100 and 250. During this period, Ebola virus and Zika virus outbreaks in West Africa broke out and had a great impact on the outbreak area, which further strengthened the scholars’ attention on researching PHE and produced more research results. From 2019 to 2020, the number of publications increased dramatically, from 207 in 2019 to 964 in 2020. Due to the COVID-19 pandemic in 2019, scholars in various countries have greatly improved their research and attention to PHE. The research on public health events reached the climax of the ancient Chinese literature search. With the input of public health services, PHE will be more and more important. The research in this field will also be more extensive and profound.

#### 3.1.2. Co-Author Analysis

**Figure 2:**
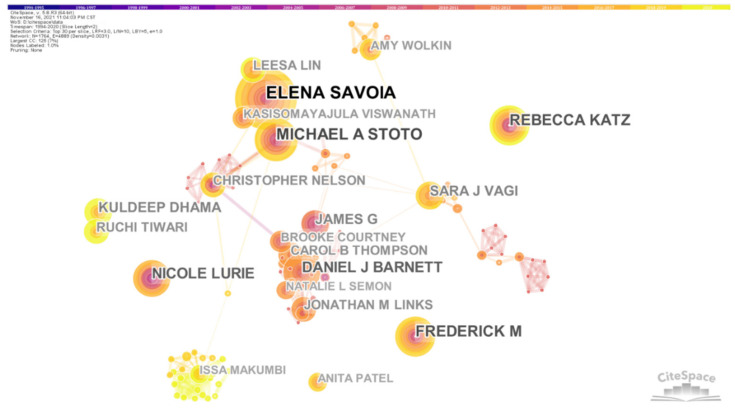
Co-authorship network (1994–2020). Each node corresponds to an author. The larger size of the node, the more papers the author has collaborated to publish. The thicker the line between the nodes, the closer the two authors work together.

Analyzing international author collaborative networks of knowledge mapping, we can clearly see the core authors in the PHE field. Figure 2 was obtained by analyzing the cooperative network of authors, with a total of 751 nodes and 1024 links. The nodes are relatively scattered, and there are few connections between nodes. This shows that although PHE researchers have a certain scale, they are classified, and they have many relatively isolated researchers. The most prolific authors are from Johns Hopkins University, Harvard University and other academically active universities.

Table 1 lists the top seven core authors with a large number of publications, including Elena Savoia (18 published pieces of literature), Daniel J Barnett (12), Michael A Stoto (10), Nicole Lurie (9), etc. The most prolific contributor in the public health emergency of international concern research field is Elena Savoia. Dr. Savoia’s professional field is public health emergency preparedness. She is one of the early scholars who began to study public health system emergency preparedness. Her research in the field of public health has a certain authority. In Figure 2, Elena Savoia and her co-author Michael A Stoto had focused on public health emergencies of international concern for a long time, and there were many cooperative research articles from 2000 to 2010. From 2010 to now, the research is still deepening. They reviewed the relevant literature in the field of public health emergency preparedness [22], concluded that most studies lacked strict design, questioned the effectiveness of the results and proposed a conceptual framework in 2017 to describe the basic elements to be considered when applying performance measurement science to public health emergency drills [23]. This provides important theoretical significance for practitioners and researchers who hope to better test the efficiency of emergency drills and improve the level of public health emergency response. Meanwhile, Savoia also participated in the research on the public health emergency preparedness system, which evolved from a general investigation to an analysis of specific intervention measures, and conducted more empirical research, providing evidence basis for public health practitioners [24].

#### 3.1.3. Co-Institution Analysis

**Figure 3:**
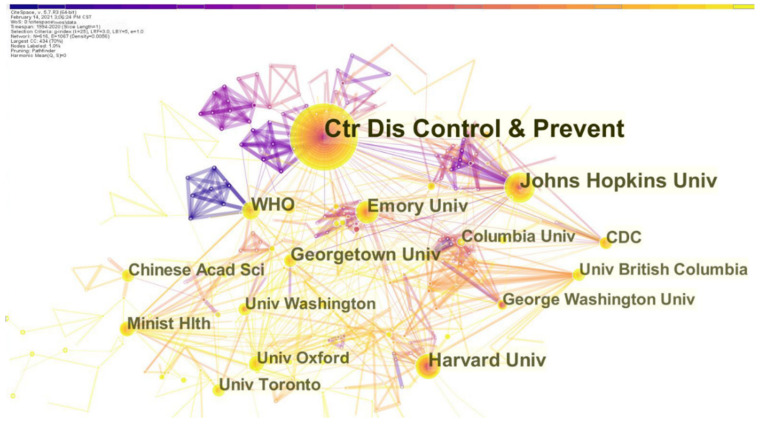
Co-institution network (1994–2020). Different colors represent different time slices, the larger the node, the more papers institutions have published. The thicker the line between the nodes, the closer the two institutions work together.

We ran the CiteSpace to generate a network as usual: 1994–2020, Slice length: 1 year; Node Select the node type: Institution, appropriate Top N was adjusted, choice Pathfinder and Pruning the merged network. Other parameters were the default settings. The map was generated, as shown in Figure 3, with 616 nodes and 1067 links in total. Figure 3 shows the co-institution cooperative network research map in the field of PHE. The overall color of the co-institution cooperation network diagram is warm, and the connecting lines are both yellow and purple. This shows that there are still exchanges and cooperation among institutions. Due to the outbreak of COVID-19 since the end of 2019, this is a common difficulty and problem faced by the world, and international exchanges and cooperation are closer.

From the perspective of institutions in terms of publications, there are nine institutions with a number of publications of 30 or more, as shown in Table 2. The Centers for Disease Control and Prevention (CDC) ranks first in the number of publications, with 185 papers in total. It was followed by Johns Hopkins University, Harvard University, The World Health Organization, Emory University, Georgetown University, the University of Toronto, the Chinese Center for Disease Control and Prevention and the Chinese Academy of Sciences.

#### 3.1.4. Co-Country/Region Analysis

**Figure 4:**
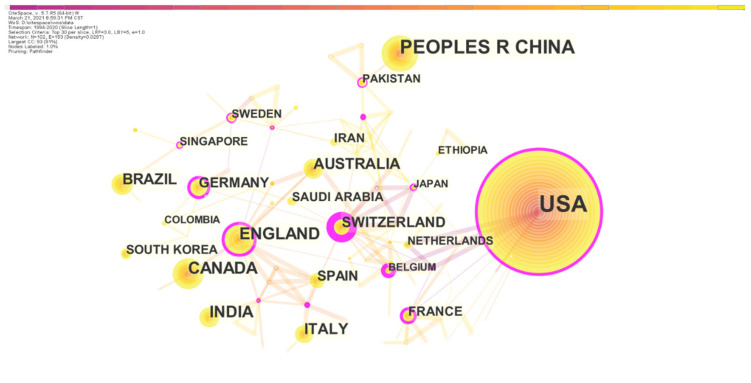
Co-Country/Region network (1994–2020). A node corresponds to a country or region. The larger the node, the more papers a country or region has published. The thicker the line between the nodes, the closer the two countries or regions work together.

We ran the CiteSpace, generating a network as usual: 1994–2020, Slice length: 1 year; Node Select the node type: Country, Top N = 30, choice Pathfinder and Pruning the merged network. Other parameters were the default settings. Co-Country/Region networks were generated with a total of 102 nodes and 367 lines. In Figure 4, the distribution of each region is relatively concentrated and closely related to each other. According to the node size, the USA published the most papers on PHE, with a total of 1210 papers. In second place was China, which published 371 English articles, followed by the UK (174), Canada (155), India (138), Brazil (101), etc.

From the centrality (see Table 3), Switzerland has the highest central value, with a central value of 0.87, which is much higher than other countries/regions. It plays an important intermediary role in promoting the integration and exchange of international PHE research achievements. Although the number of research articles issued by Switzerland is far less than that of the United States, China and other countries, Switzerland plays the most obvious intermediary and key, pivotal role in the research field of PHE and has great influence and more exchange with other countries. This is because Switzerland’s medical and health care services are very developed, and the management system of medical institutions is sound. After continuous reform and improvement, it has characteristics of the symbiosis of medical institutions with multiple ownerships and the coexistence of multi-level medical organizations and has formed a relatively perfect medical and health system suitable for the market economic system [25]. The second is the UK (0.38), with a small number of publications and a large centrality, which shows that the UK had a certain influence and strong intermediary role in this field. The USA (0.29) ranks third and has a high centrality. In Figure 4, the USA has a purple outer ring outside the node, indicating that it is between the centers. Therefore, it can be considered that the USA plays an important role in inter-agency research cooperation. In addition, as the country with the largest number of publications, the USA has a large number of studies in the PHE research field, which has considerable influence. In addition, it can be observed that China has the second-largest number of publications, but the centrality is less than 0.01. The PHE research literature was published for the first time in 2003. It can be seen that there are a large number of publications and more research on PHE, but China pays late attention to this field, has insufficient influence, weak intermediary role and lacks communication and cooperation with various countries and regions. Compared with other countries, there is still a gap in the research of PHE, which can further strengthen international exchanges and cooperation and speed up the development of public health in China.

### 3.2. Research Hotspot and Frontier Evolution Analysis

#### 3.2.1. Keyword Co-Occurrence Analysis

**Figure 5:**
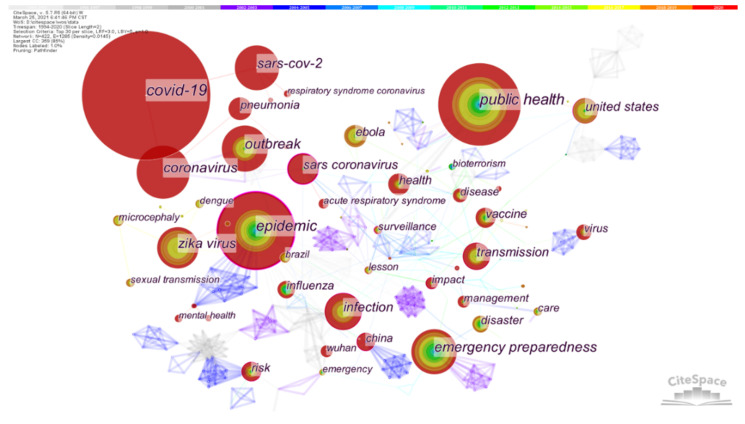
Co-occurrence network (1994–2020). A node corresponds to a keyword. The larger the node, the higher the frequency of keyword reference. The ring around the node represents the frequency of keyword occurrence in different years, and the color of different depths represents different years. The thickness of the ring is directly proportional to the frequency of the word in that year, and the line between nodes represents the degree of connection between keywords.

We ran the CiteSpace to generate the keyword co-occurrence map and to analyze the hot spots of PHE: 1994–2020, Slice length: 2 years; Node Select the node type: Keyword, Top N = 30, choice Pathfinder and Pruning the merged network. Other parameters were the default settings. Co-Country/Region networks were generated with a total of 422 nodes and 1503 lines. In Figure 5, the high-frequency keywords in this field are COVID-19, Public Health, Epidemic, and Coronavirus with frequencies of more than 200 times, and Emergency Preparedness, Outbreak, SARS-CoV-2, Zika virus, Infection, SARs coronavirus, United states, Transmission and Health with frequencies of more than 100 times.

From the frequency and centrality of keywords (see Table 4), current international research hotspots of PHE mainly include COVID-19, public health, epidemic, emergency preparedness, disease transmission, United States, model, system, pneumonia, vaccine, influenza, management, surveillance, impact, care and so on. The high-frequency keyword with the highest centrality is “epidemic”, reaching 0.41. Followed by “monitoring” (0.33), “epidemic degree” (0.22), “SARs virus” (0.22), “model” (0.19), “system” (0.15), “United States” (0.14), “children” (0.14), “emergency response” (0.14), “public health preparation” (0.14), “communication” (0.14) and other keywords. In the field of PHE, the research on the epidemic situation of major infectious diseases accounts for a large proportion and has a great impact on society. The Middle East respiratory syndrome coronavirus, Ebola virus, measles and Zika virus have always been public health events of international concern. Scholars also pay more attention to the research on epidemics, so epidemics have a high intermediary centrality and a strong intermediary role. These keywords play a greater intermediary role and are more closely related to other content in the PHE field.

According to Table 4, as a keyword appearing for the first time in 2020, COVID-19 has a frequency of 486 times, ranking in first. Its research output is in a period of rapid growth. In 2019, the outbreak of COVID-19, a global pandemic, became the sixth public health emergency of international concern declared by the World Health Organization. COVID-19 has become the focus of research from all walks of life, which has greatly increased people’s attention to PHE and set off a research boom in the field of public health events. Since 2019, the number of publications in the PHE field has increased rapidly, and the impact of COVID-19 continues. Although related keywords, such as “COVID-19” and “coronavirus”, appeared late for the first time, it is reasonable for them to become the top hot words.

#### 3.2.2. Burst Keywords Analysis

**Figure 6:**
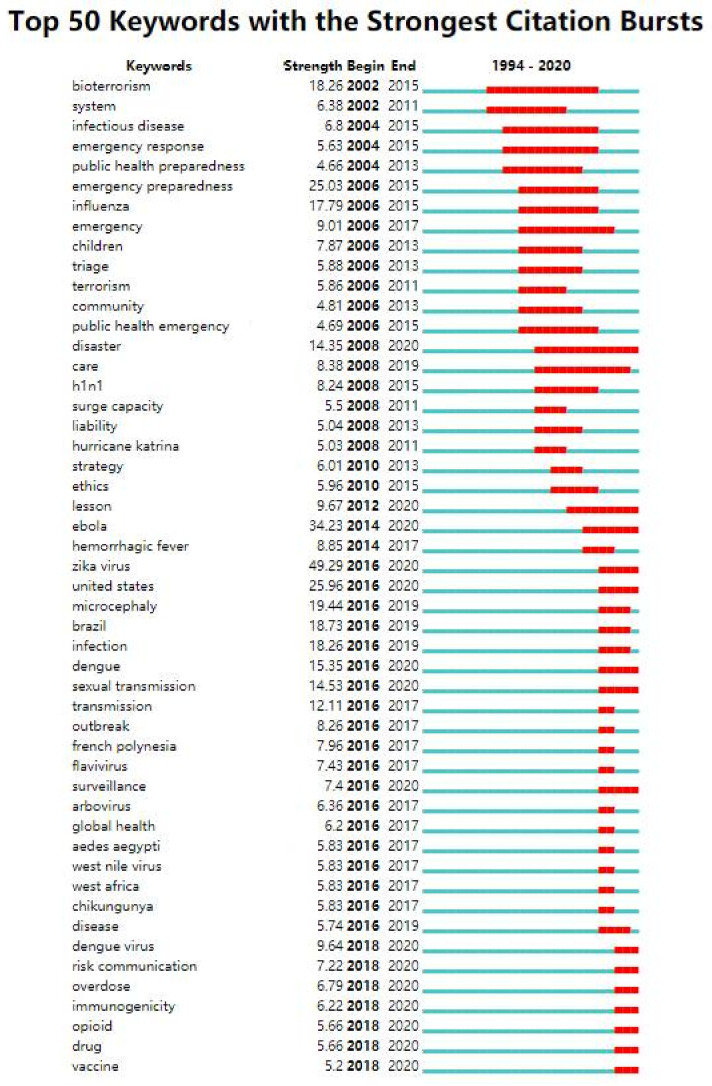
Top 50 keywords with strongest citation bursts (sorted by emergent time).

Burst detection analysis can identify and explore the research frontier and latest trends in a certain field. According to the selection of burst nodes, it can be divided into burst keywords, documents, authors, journals and fields. Burst words refer to the keywords with a sudden increase in the number of citations or occurrences in a certain period. Its basic principle is to determine the hot words according to the growth rate of keyword occurrence frequency. The correlation characteristics between these hot words and time are usually regarded as the research frontier in a certain field [26]. With a keyword burst detection analysis of CiteSpace software, a total of 61 burst keywords were found in the PHE field. Figure 6 shows the situation of the top 50 keywords with the strongest citation bursts (sorted by emergent time). In terms of burst intensity, the top five burst keywords were “zika virus” (49.29), “Ebola” (34.23), “United States” (25.96), “emergency preparedness” (25.03) and “microcephaly” (19.44).

According to the burst time and duration of keywords, the hot frontiers of PHE in different years were analyzed. In Figure 6, burst keywords from 2002 to 2012 is overlapped and continued until 2015, indicating that “infectious diseases”, “flu”, “emergency response”, “emergency preparedness” and “biological terrorism” were hot spots for scholars in the past 10 years, involving children, society, medical care, ethics, care and other aspects of research. From 2014 to 2016, there were many burst words related to viruses and diseases, such as Ebola virus, Hemorrhagic fever, Zika virus, Microcephaly, Dengue fever, French Polynesia, Flavivirus, Arbovirus, West Nile virus, Chikungunya, etc. This is the period with the most burst words in two years. A total of seven burst keywords appeared in 2018, and “opioid”, “overdose”, “vaccine”, “immunogenicity” and “risk communication” became new research hotspots. These burst words appeared late in the year but with a high burst intensity, and the burst time lasted until 2020. In terms of the end time of a burst, the burst time of disaster, United States, Zika virus, Ebola, Dengue, Surveillance, Care, Lesson, Sexual transmission and other words with earlier burst years also continued until 2020, and the burst intensity was also high.

The emergence and continuation of burst keywords are closely related to the outbreak and the impact of the epidemic. Up to now, all kinds of serious infectious diseases are still happening in the world. In August 2018, the 10th Outbreak of Ebola in The Democratic Republic of the Congo (DRC) was declared a public health emergency of international concern by WHO on 17 July 2019 [27]. WHO warned that the Ebola virus exists in animal hosts in this region and can persist for several months in some body fluids of survivors. There is still the possibility of secondary transmission, so it is necessary to maintain vigilance and strong surveillance [28]. In 2020, The Americas and Southeast Asia became the two regions where dengue fever occurred frequently. Brazil has reported the most dengue cases in the world, accounting for 63.4% of the total cases in the Americas [29]. Meanwhile, the number of Aedes aegypti, the main vector of dengue fever, also increased after monitoring. To some extent, the outbreak of COVID-19 has exacerbated the dengue fever [30,31,32]. Similarly, in 2020, about 14,000 Zika virus cases were reported in South America, with the largest number in Brazil, with a cumulative report of 12,893 cases, accounting for 90%. Due to the international public health events in recent years, the current international important infectious disease events are consistent with the frontier hotspots, such as “Zika virus”, “Ebola virus”, “dengue fever”, “epidemic monitoring” and “transmission route” analyzed in this paper.

#### 3.2.3. Research Frontier Evolution—Timeline Analysis

**Figure 7:**
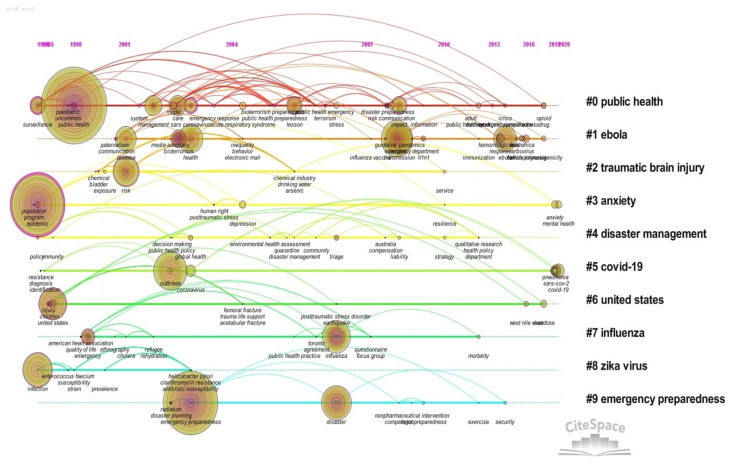
Timeline chart.

The timeline chart can reflect the temporal evolution of keywords under a cluster, contributing to the analysis of the evolution trend of the research frontier in the development process of PHE. The two indexes Q = 0.8747 and S = 0.9526 to measure the clustering effect indicate that the formed clustering structure is significant and the clustering is reasonable. In Figure 7, the research frontier of PHE abroad is mainly reflected in the following keyword clusters.

#0 Public health. From 1994 to 1998, there appeared two big nodes of surveillance and public health. In the early stage, international attention was paid to the surveillance research of PHE. Then, from 2001 to 2005, high-frequency keywords, such as “system”, “management”, “Sars coronavirus”, “model”, “coronavirus” and “system”, appeared. The research hotspots have evolved to SARS coronavirus, system model construction in PHE, emergency system management, care for children, experience learned in emergency response and so on. From 2006 to 2008, emotional stress, risk communication and impact in public health emergencies became the focus of attention. In 2018, the keywords of opioids appeared for the first time, and scholars began to pay attention to the study of the opioid crisis [33].

#1 Ebola. From 2001 to 2004, “disease”, “health” and “bioterrorism” appeared for the first time and bioterrorism, public health emergency preparedness and public health have become the research frontier. From 2008 to 2009, the research hotspots gradually shifted to the transmission route of the disease, vaccine research and development, the H1N1 influenza epidemic situation and so on. From 2014 to 2018, the Ebola epidemic occurred during this period, and research on Ebola virus, hemorrhagic fever, emergency response, French Polynesia, arbovirus, West Africa, immunogenicity and other aspects became hotspots, involving the virus itself, origin, transmission route and vector, immune mechanism and other aspects [34].

#3 Anxiety. This node of epidemic appeared for the first time in 1994, “depression” appeared in 2004, and emotional keywords such as “mental health” and “anxiety” appeared in 2020. In the context of PHE, the influence and treatment of epidemic-related anxiety disorder in mental health is a research hotspot.

#4 Disaster management. There are a few pieces of research literature in this cluster. In 2003, a large node, “Global health”, appeared for the first time. Scholars studied public health disaster plans from the perspective of global health security and health [35]. In 2006, the keyword “triage” appeared, and the allocation model of scarce resources in disasters and the classification method for the decision-making process became the research hotspot [36].

#5 COVID-19. In this cluster, the keywords “outbreak” and “coronavirus” first appeared in 2003, and the characteristics of SARs coronavirus were discussed. There were no new keywords for a long time until the outbreak of COVID-19 in 2019. High-frequency keywords such as “COVID-19”, “SARS-CoV-2” and “pneumonia” appeared in 2020. The research on COVID-19 had attracted extensive attention from international scholars. On the one hand, scholars have carried out studies on its origin, pathogenesis, genetic variation, immune response, vaccine development and other aspects from the perspective of the genetic composition of novel coronavirus. On the other hand, scholars paid attention to the research on people’s psychological stress during the outbreak of COVID-19 [37], the construction of a psychological crisis intervention model, the prediction model of PHE, risk communication, emergency preparedness and planning, etc. As the first country to take up COVID-19, “China” and “Wuhan” were also frequently used as keywords.

#6 United States. “United States” and “children” first appeared in 1996 and had produced a certain amount of research output, including the study of American epidemic diseases, the study of the challenges of childhood patients’ diseases in disasters, the study of health information systems for children, and the legal issues related to children in PHE. Then, in 2016, the West Nile virus appeared, and the related Zika virus got attention and became a research hotspot. In 2018, the keyword “overdose” appeared, and the study of PHE caused by the opioid overdose crisis in the United States attracted the attention of scholars.

#7 Influenza. In 1999, “emergency” appeared as a large node involving the study of PHE and emergency response. In 2006, the keyword “influenza” appeared, and scholars conducted research on many pandemics, such as H1N1 influenza [38].

#8 Zika virus. This clustering study mainly shows and discusses the epidemic situation of the Zika virus. “Infection” appeared for the first time in 1994, and high-frequency keywords, such as “Zika virus”, “microcephaly”, “Brazil”, “dengue” and “sexual transmission”, appeared in 2016 [39,40]. Zika virus’s research on infection prevention, transmission route and vector, and the flow of microcephaly in Brazil have become research hotspots in this field [41].

#9 Emergency preparedness. The research on emergency preparedness for PHE has increased year by year, and the research heat continues to be high. The research in this cluster focuses on crisis risk communication in emergency preparedness, community resilience, training plans to improve emergency preparedness, framework of information system, disaster risk information assessment, thoughts caused by bioterrorism and pandemic influenza, and so on [42,43].

## 4. Conclusions

This study used CiteSpace 5.7 to analyze 2247 research articles using the scientometric method and discussed the research status and development trends of PHE. The main conclusions are as follows.

At present, the outbreak of COVID-19 draws more researchers’ attention to PHE. As a research topic of international concern, the United States is in the leading position, and China and England also have significant places in this field. Switzerland has the highest central value and plays an important intermediary role in promoting the integration and exchange of international PHE research achievements.

The main research hotspots and evolutionary context of PHE focused on COVID-19, public health, epidemiology, coronavirus, emergency preparedness, transmission routes, etc., with emphasis on international researches and many mature academic achievements. In addition, COVID-19 has become the focus of research from all walks of life, and its impact will continue for a long time.

The cooperation and exchange between various institutions are frequent, but the problem of imbalance distribution and less cooperation of high-yield researchers still persists. With the development of globalization, international communication and cooperation between authors may be strengthened.

The intermittent occurrence of international emergency public health events promotes the development of research in this field: the germination of research from 1994 to 2003 until the outbreak of SARS in 2003 pushed the first outbreak of research in this field (2004–2014). The emergence of the Ebola virus and Zika virus further strengthened scholars’ attention on researching public health emergencies (2015–2019). Since the end of 2019, the global outbreak of COVID-19 has pushed PHE research to the center of the international arena. The research on PHE in various countries has reached an unprecedented climax. In the future, the research on emergency preparedness and disaster management will still be active fields, and how to effectively prevent major public health emergencies will continue to draw attention.

## Figures and Tables

**Table 1:** The top 7 authors in terms of publications.

Code	Number of Publications	Centrality	Year of First Occurrence	Authors
1	18	0.00	2009	Elena Savoia
2	12	0.00	2009	Daniel J. Barnett
3	10	0.00	2009	Michael A. Stoto
4	9	0.01	2007	Nicole Lurie
5	8	0.00	2014	Sara J. Vagi
6	8	0.00	2017	Kuldeep Dhama
7	8	0.00	2008	Frederick M.

**Table 2:** The top 10 institutions in terms of publications (30 times or more).

Code	Number of Publications	Centrality	Percentage of Total Publications	Institutions	Country
1	185	0.41	8.23	Ctr. Dis. Control & Prevent	USA
2	77	0.12	3.43	Johns Hopkins Univ.	USA
3	53	0.11	2.36	Harvard Univ.	USA
4	44	0.10	1.96	WHO	—
5	40	0.04	1.78	Emory Univ.	USA
6	39	0.02	1.74	Georgetown Univ.	USA
7	33	0.13	1.47	Univ. Toronto	Canada
8	32	0.03	1.42	CDC	Peoples R China
9	32	0.08	1.42	Chinese Acad. Sci.	Peoples R China
10	30	0.03	1.34	Minist. Hlth	—

**Table 3:** The top 12 countries/regions in terms of publications (sorted by centrality).

Code	Number of Publications	Centrality	Country
1	72	0.87	Switzerland
2	174	0.38	England
3	1210	0.29	USA
4	53	0.29	France
5	58	0.24	Germany
6	155	0.04	Canada
7	138	0.04	India
8	101	0.04	Brazil
9	100	0.04	Italy
10	55	0.02	Spain
11	371	0.00	Peoples R China
12	101	0.00	Australia

**Table 4:** Glossary of hot topics in the PHE field.

Code	Frequency	Centrality	Keywords
1	486	0.02	COVID-19
2	331	0.10	public health
3	308	0.41	epidemic
4	201	0.05	coronavirus
5	193	0.10	emergency preparedness
6	188	0.01	outbreak
7	169	0.02	SARS-CoV-2
8	163	0.06	zika virus
9	152	0.13	infection
10	131	0.22	sars coronavirus
11	115	0.14	united states
12	114	0.14	transmission
13	102	0.03	health
14	96	0.01	ebola
15	95	0.02	vaccine
16	89	0.01	pneumonia
17	86	0.12	risk
18	81	0.08	influenza
19	80	0.03	disaster
20	73	0.1	china
21	68	0.06	virus
22	68	0.08	disease
23	58	0.02	management
24	57	0.11	impact
25	52	0.33	surveillance
26	51	0.01	care

## Data Availability

Not applicable.
